# Independent component analysis of resting-state fMRI identifies regions associated with seizure freedom after laser interstitial thermal therapy for temporal lobe epilepsy

**DOI:** 10.3389/fneur.2025.1675066

**Published:** 2025-11-25

**Authors:** Mahdi Alizadeh, Jingya Miao, Caio M. Matias, Kevin J. Hines, Christopher T. Skidmore, Michael R. Sperling, Joseph I. Tracy, Ashwini Sharan, Chengyuan Wu

**Affiliations:** 1 Department of Neurosurgery, Thomas Jefferson University, Philadelphia, PA, United States; 2 Department of Neurology, Thomas Jefferson University, Philadelphia, PA, United States

**Keywords:** temporal lobe, epilepsy, LITT, resting state fMRI, seizure freedom

## Abstract

**Objective:**

Temporal lobe epilepsy (TLE) is a common form of drug-resistant epilepsy often treated with surgical interventions, including laser interstitial thermal therapy (LITT). However, patient-specific factors influencing LITT outcomes remain unclear. This retrospective study aimed to identify pre-operative resting-state functional MRI (rs-fMRI) patterns associated with seizure freedom following LITT in mesial TLE.

**Methods:**

We analyzed rs-fMRI data from 28 patients with mesial TLE who underwent LITT, classifying them into seizure-free (SF) and not seizure-free (NSF) groups based on 12-month post-operative outcomes. Independent component analysis (ICA) was used to identify subject-specific brain networks, and generalized linear models (GLM) were employed to assess associations between pre-operative spatial patterns of ICA-derived functional connectivity (FC) and surgical outcomes, controlling for clinical variables.

**Results:**

Significant differences in brain ICA-derived FC patterns were observed between SF and NSF groups, with SF exhibiting more locally distributed ICA-derived FC patterns around the mesial temporal lobe, including the posterior orbitofrontal cortex (OFC) and anterior parahippocampal gyrus (PHG). In contrast, NSF demonstrated more diffusely distributed ICA-derived FC patterns encompassing the insula and thalami.

**Significance:**

These findings highlight the potential of pre-operative rs-fMRI as a prognostic tool for identifying TLE patients more likely to benefit from LITT. Further validation in larger cohorts is warranted to confirm these results and optimize patient selection for surgical interventions.

## Introduction

Temporal lobe epilepsy (TLE) is the most common type of focal epilepsy in adults and the most common type of drug-resistant epilepsy referred for surgical treatment (1, 2). Although anterior temporal lobectomy (ATL) remains the gold standard surgical approach, laser interstitial thermal therapy (LITT) has emerged as a minimally invasive alternative for mesial TLE (mTLE). LITT enables targeted ablation of deep mesial structures such as hippocampus and amygdala with the advantages of shorter hospital stays, fewer post-operative complications, and better preservation of cognitive function (3–6). The overall seizure-freedom rates following LITT has been reported from approximately 56% at 1-year follow-up to 53% at 2-years follow-up (7).

Despite growing clinical use, predictors of LITT outcomes remain inconsistent across studies. Some studies have reported that neither the presence of mesial temporal sclerosis (MTS) nor the ablation volume was significantly correlated with improved seizure outcomes (3, 5, 8), whereas others have found improved post-LITT seizure outcome among patients with radiographically evident MTS (9–11). These discrepancies may resulted from variations in imaging criteria, surgical targeting, small sample size, or patient selection. In contrast, the location of ablation has shown more consistent associations with favorable outcomes particularly when the amygdala, hippocampal head, parahippocampal gyrus (PHG), piriform cortex, and rhinal cortices are effectively included within the ablation zone (3, 12).

TLE is increasingly recognized as a network disorder rather than a purely focal pathology and characterized by neural dysfunction extending beyond the identified epileptogenic zone (EZ) (13, 14). Surgical failure after LITT may therefore reflect the persistence of pathological connectivity extending beyond the ablated mesial structures into broader extratemporal regions (15–17). Identifying the network patterns associated with surgical success may provide novel biomarkers for patient selection and prognostication.

Independent component analysis (ICA) offers a data-driven approach to mapping intrinsic functional connectivity (FC) patterns, enabling identification of networks that may underlie seizure outcomes (18). We hypothesized that ICA-derived network patterns would differ between patients who achieved seizure freedom (SF) and those who did not (NSF) following LITT for mTLE, reflecting distinct functional architectures related to surgical efficacy.

## Methods

### Patient selection

This study was approved by the Institutional Review Board and exempted from obtaining informed consent due to the retrospective nature of the study. Patients with drug-resistant epilepsy who underwent LITT at our institution from March 2012 to June 2018 were first identified. All patients underwent standard pre-operative evaluation by a multidisciplinary team including neurologists, neurosurgeons, neuropsychologists and neuroradiologists.

Inclusion criteria included a diagnosis of mTLE (based on seizure semiology, pre-operative structural T1-weighted MRI, positron emission tomography (PET) scans, and long-term video-EEG monitoring), pre-operative rs-fMRI, LITT targeting mesial temporal structures including the amygdala and hippocampus, and a minimum 12-month follow-up with documented seizure outcomes. Although radiographically confirmed dual pathology was not found to be associated with LITT outcomes (3), it has been implicated in contributing to seizure recurrence following temporal lobectomy (19). Given the intricate influence of additional pathologies on resting-state data, patients with seizure etiologies other than mTLE were excluded. Additional exclusion criteria included the need for repeat procedures or the absence of pre-operative rs-fMRI.

Seizure outcomes were obtained from medical records and were categorized as either SF or NSF. Patients who had no seizures or experienced only auras 12 months after LITT (Engel Class Ia and Ib) were classified as SF; those who experienced any type of seizure post-operatively were classified as NSF. Clinical information, including age at disease onset, gender, age at the time of procedure, presence of pre-operative tonic-clonic seizures, and laterality of disease, were also obtained from medical records.

### Image acquisition

All patients underwent imaging acquisitions prior to their surgery using a 3.0T Achieva Phillips scanner with an eight-channel head coil. T1-weighted imaging was obtained (FOV = 24.0 cm, voxel size = 1.0 × 1.0 × 1.0 mm^3^, matrix size = 512 × 512, TR = 12 ms, TE = 6 ms, and slice thickness = 1 mm). Then, participants were instructed to relax, keep their eyes open, and avoid specific thoughts for a total duration of 12 min. rs-fMRI scans were acquired axially using a single-shot echo planar imaging sequence (FOV = 23.0 cm, voxel size = 3.0 × 3.0 × 3.0 mm^3^, matrix size = 128 × 128, TR = 2.5 s, TE = 26 ms, number of averages = 1).

### Image processing and ICA

To maintain consistency in the analysis regarding the ipsilateral and contralateral sides in TLE, images from patients who underwent right-sided LITT were flipped right to left. As such, the left side of each image represents the hemisphere ipsilateral to mTLE; and the right side represents the contralateral hemisphere.

All rs-fMRI data underwent pre-processing and ICA using the MELODIC toolbox in FSL (FMRIB Software Library, https://fsl.fmrib.ox.ac.uk/fsl/fslwiki/FSL) (20). Pre-processing included motion correction, slice timing correction, spatial smoothing (FWHM = 5 mm), and low-pass temporal filtering (0.01 Hz). Single-session ICA was then carried out to generate subject-specific independent components (ICs). Voxels contributing to each component were identified using MELODIC's mixture model-based inference, with the default threshold level set to 0.5. The final output consisted of thresholded spatial maps with voxel intensities expressed as *z*-scores. The dimensionality of decomposition was automatically estimated by MELODIC based on the Laplace approximation to the Bayesian evidence for different model orders, ensuring that the number of ICs was optimized for each dataset given its number of timepoints.

Note that, all fMRI data were spatially normalized to MNI152 standard space using FSL's non-linear registration tools (FNIRT). Specifically, each subject's functional images were first aligned to their structural T1-weighted image using boundary-based registration (BBR), and then the structural image was non-linearly warped to MNI space. The transformation parameters were applied to the IC spatial maps to ensure that all components were analyzed in a common MNI space.

**Figure 1:**
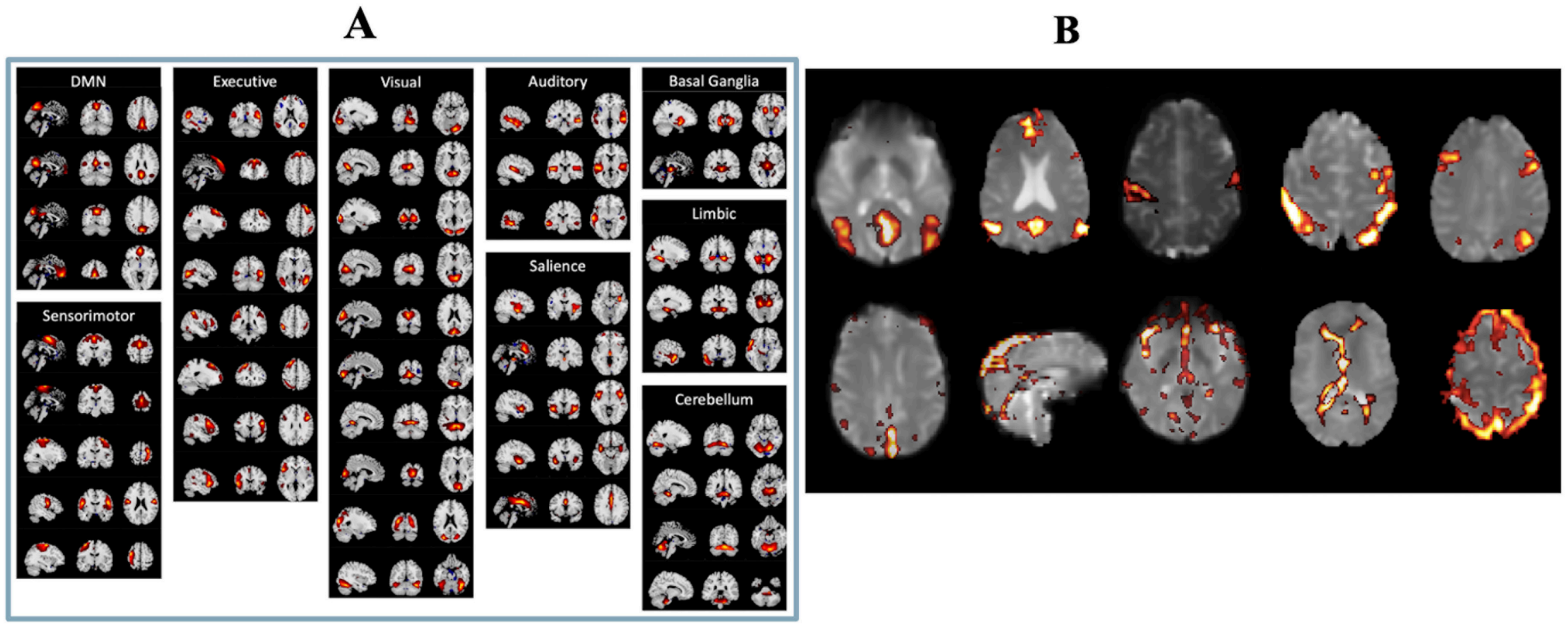
**(A)** List of real resting state functional networks. **(B)** Examples of independent components considered to be real (top row) and independent components considered to be artifactual (bottom row).

After ICA, two authors (CMM and MA) manually inspected the ICs, following the selection guide outlined by Boerwinkle et al. (21) to distinguish meaningful components from noise components. Both authors were blinded to both the patients' surgical outcomes and LITT laterality at the time of component selection to minimize potential bias. Components were classified based on combined evaluation of spatial maps, temporal dynamics, and frequency spectra, with reference to canonical resting-state network templates. Components showing edge artifacts, head motion, high-frequency noise (>0.1 Hz), or physiologic/vascular signal contamination were excluded. ICs were deemed meaningful if they exhibited peak FC patterns in known resting state networks (RSNs), gray matter, or the brainstem, had power spectra predominantly in the low-frequency range, and showed time courses characterized by slow, regular oscillations. Representative examples of both included and excluded ICs are shown in Figure 1. To minimize any potential ambiguity related to the arbitrary sign of ICA-derived *z*-scores, we applied a positive threshold of *z* > 2.5 to identify meaningful components. Only components with positively weighted voxels exceeding this threshold were retained. We did not observe any meaningful resting-state networks with significant negative *z*-scores (*z* < −2.5), ensuring that all selected components reflected positive connectivity patterns relative to the global mean.

### GLM and statistical analysis

A voxelwise general linear model (GLM) was implemented using custom MATLAB scripts to examine the relationship between pre-operative IC spatial patterns and surgical outcomes. The dependent variable (Y) was surgical outcome (SF = 1, NSF = 0). Each selected IC spatial map was treated as an independent predictor. Because multiple ICs were derived from the same subject, subject identity was included as a covariate (dummy-coded) to account for repeated measures within individuals and to reduce bias related to within-subject dependencies.

Model fitting was performed using MATLAB's *fitlm* function. For each voxel, the model estimated β coefficients, standard errors, *t*-statistics, and *p*-values for each predictor. This implementation follows a fixed-effects GLM framework with subject identity included as covariates, rather than a full mixed-effects model with estimated random effects. Additionally, several clinical covariates (Xi) were included in the model: age at surgery, gender, age at epilepsy onset, and presence of tonic–clonic seizures prior to surgery. Contrasts were applied to the β estimates associated with the ICA *z*-score predictor to identify regions where greater connectivity strength was positively or negatively associated with seizure freedom (β > 0 for SF > NSF, β < 0 for NSF > SF). Voxelwise *t*-statistic maps were generated for contrasts of interest (SF > NSF and NSF > SF). Significant clusters were identified using false discovery rate correction (*p* < 0.05) with a minimum cluster extent of 20 contiguous voxels (22). These clusters represent pre-operative rs-fMRI ICA-derived functional connectivity patterns predictive of post-surgical outcomes.

**Figure 2:**
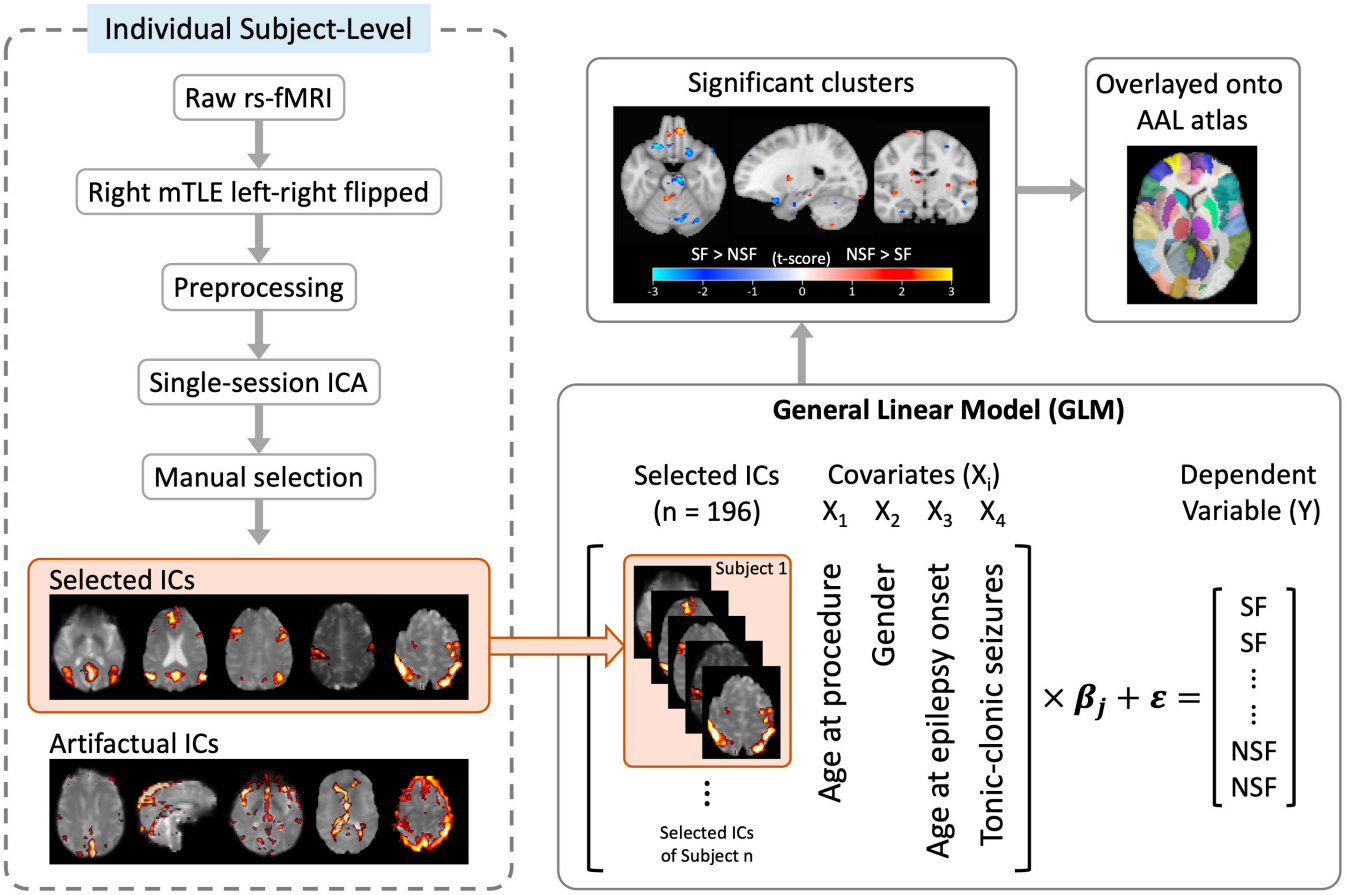
Flowchart of image processing and analysis. ICA, independent component analysis; ICs, independent components.

Finally, significant clusters were overlaid onto the AAL atlas (23) to identify the involved anatomical regions. A pipeline of the image processing and analysis workflow is illustrated in Figure 2. Demographic and clinical characteristics between groups were compared using the Mann–Whitney and Chi-square tests as appropriate.

## Results

### Patient characteristics and ICA results

A total of 28 patients met our inclusion criteria. At the 1-year post-operative follow-up, 17 (60.7%) patients achieved seizure freedom (Engel Class Ia and Ib) and were included in the SF group, while 11 (39.3%) patients were classified as the NSF group. There were no significant differences between the SF and NSF groups regarding patients' age at procedure, gender, side of TLE, presence of MTS on MRI, presence of pre-operative TC seizures, age at epilepsy onset, and duration of epilepsy. Manual IC selections after single-session ICA identified a mean of seven meaningful ICs (SD = 2.5) and 52.5 artifactual ICs (SD = 16.1) per patient. No significant differences were found between the SF and NSF groups regarding the number of selected and artifactual ICs. Patient demographics, clinical characteristics, and the number of ICs after manual selection are summarized in Table 1.

**Table 1:** Patient demographics and clinical characteristics relative to seizures.

**Group**	**Total *n* = 28**	**Seizure free (SF) *n* = 17**	**Not seizure free (NSF) *n* = 11**	***P* value**
Age at LITT (years)	52.1 ± 14.6	52.1 ± 13.4	52.1 ± 16.9	0.99
Males	12 (43%)	7 (41%)	5 (45%)	0.82
Left TLE	22 (79%)	13 (76%)	9 (82%)	0.74
MTS on MRI	26 (93%)	16 (94%)	10 (91%)	0.75
Presence of TC	17 (61%)	7 (41%)	4 (36%)	0.80
Age at onset (years)	19.6 ± 16.2	22.7 ± 15.6	14.9 ± 16.9	0.23
Duration of epilepsy (years)	32.4 ± 16.8	30.6 ± 16.9	35.3 ± 16.9	0.48
Selected ICs	7 ± 2.5	6.8 ± 1.9	7.4 ± 3.3	0.70
Artifactual ICs	52.5 ± 16.1	54.4 ± 15.2	49.5 ± 17.6	0.31

Results are presented in n (%) or mean ± SD.

TLE, temporal lobe epilepsy; MTS, mesial temporal sclerosis; TC, tonic-clonic seizures; LITT, laser interstitial thermal ablation; ICs, independent components.

### Significant clusters

**Figure 3:**
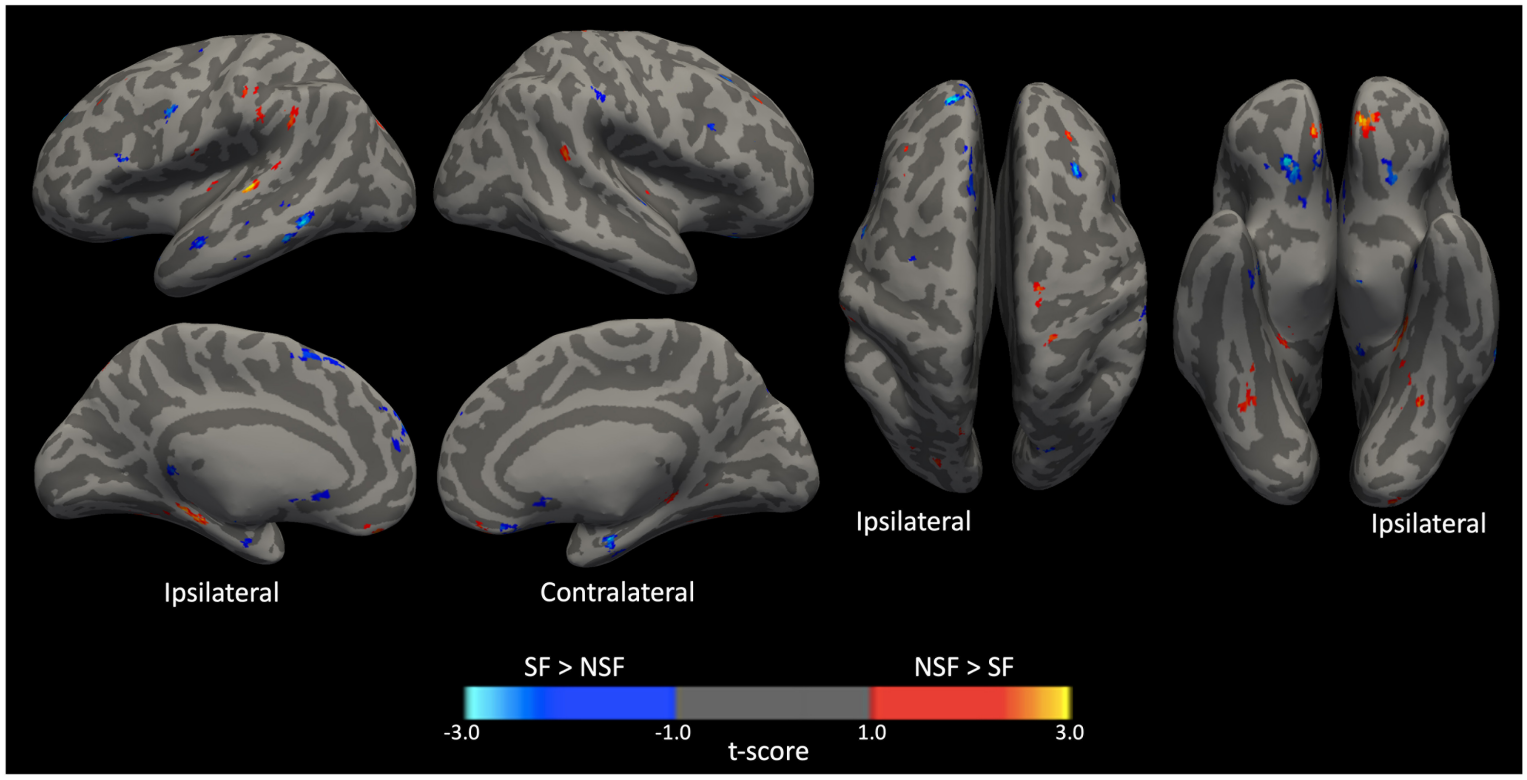
Significant clusters displayed on smoothed cerebral surface. L, left/ipsilateral; R, right/contralateral; SF, seizure free; NSF, not seizure free.

**Figure 4:**
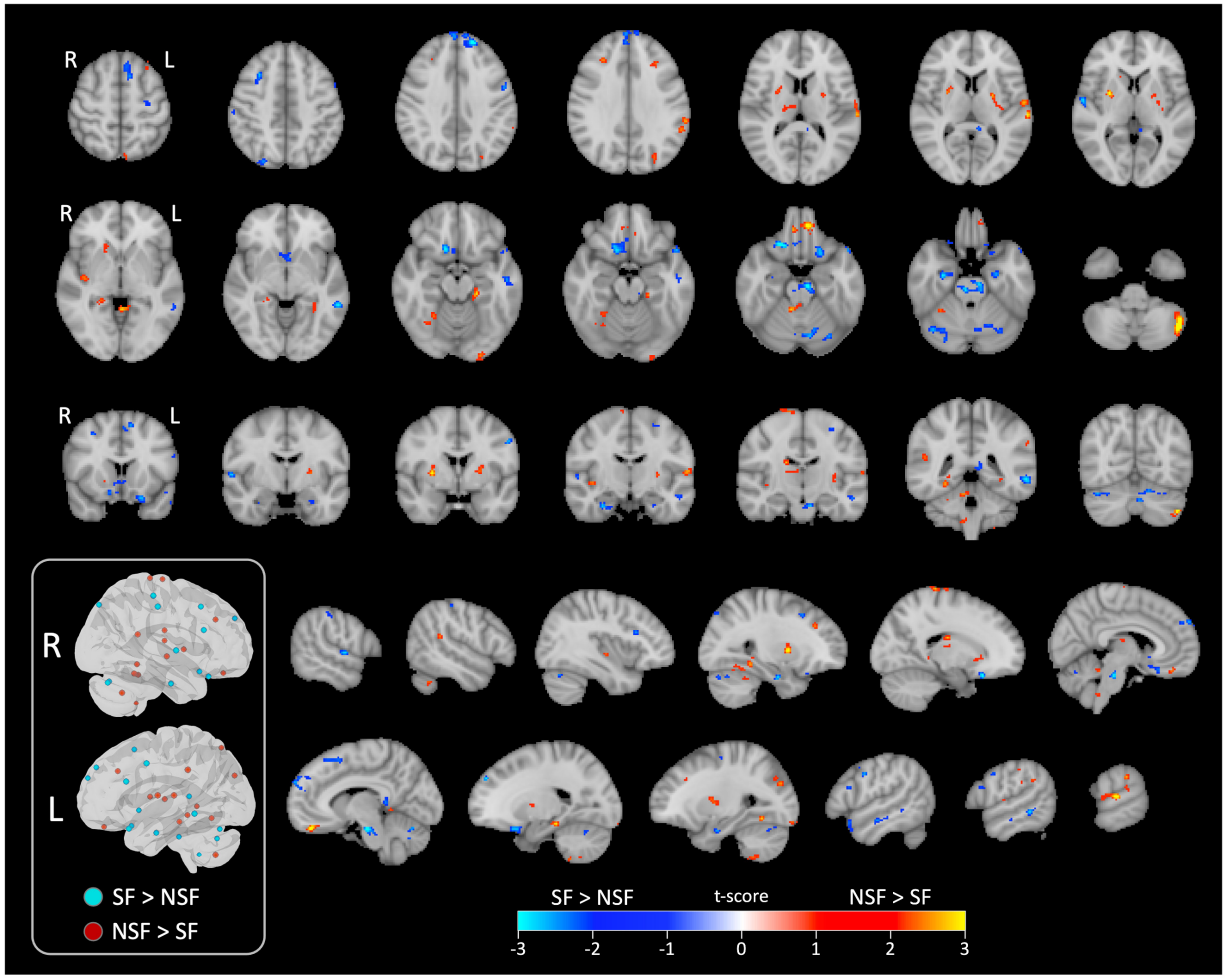
Significant clusters displayed on series of image slices. Schematic visualization of the significant clusters in 3D brain views (inside the box). All clusters that were ipsilateral to the side of TLE are shown in the left view, while contralateral clusters are shown in the right view. L, left/ipsilateral; R, right/contralateral; SF, seizure free; NSF, not seizure free.

The GLM analysis, adjusted to clinical data, yielded 24 significant clusters indicating higher brain ICA-derived FC patterns in the SF group than in the NSF group; and 34 significant clusters with higher FC pattern in the NSF group. The involved AAL brain regions of each cluster, cluster sizes, and the coordinates of the maximum *t*-score are listed in Supplementary Table S1. The major brain regions involved in clusters were visually assessed (Figures 3, 4) and summarized in Table 2.

**Table 2:** Major brain regions with higher resting state ICA-derived FC patterns associated with seizure outcomes.

**Area**	**Seizure free (SF)**		**Not seizure free (NSF)**	
Frontal	Bilateral	Posterior OFC Ventrolateral frontal	Bilateral	Anterior OFC
	Ipsilateral	SMA and Precentral	Contralateral	SMA and Precentral
Temporal	Bilateral Ipsilateral	Anterior PHG Anterior superior temporal Middle temporal gyrus	Bilateral	Posterior PHG Posterior superior temporal Fusiform
Parietal			Ipsilateral	Supramarginal
Insula and Basal ganglia			Bilateral Contralateral	Insula cortices Putamen Caudate and thalamus
Brainstem		Pons		
Cerebellum	Bilateral	Posterior cerebellum	Bilateral Contralateral	Posterior cerebellum Anterior cerebellum

In temporal lobe, the SF group had greater ICA-derived FC patterns in the bilateral anterior PHG, bilateral anterior superior temporal gyrus, and the ipsilateral middle temporal gyrus. In contrast, the NSF group exhibited greater FC patterns in the posterior and basal temporal lobe extending to the fusiform gyrus bilaterally and the ipsilateral middle occipital gyrus.

In frontal lobe, there was greater FC pattern in the bilateral basal and mesial frontal regions, including the posterior orbitofrontal cortex (OFC). The NSF group, by comparison, demonstrated greater FC pattern in the bilateral anterior OFC and contralateral supplementary motor area (SMA) and precentral gyrus (Figure 3).

Within cerebellum, both the SF and NSF groups exhibited brain FC pattern in the posterior cerebellum bilaterally. However, the NSF group had a larger network distribution, including the contralateral anterior cerebellum (Figure 4). In subcortical and insular regions, the NSF group exhibited greater FC patterns in the bilateral insula, bilateral putamen, contralateral caudate and contralateral thalamus, whereas the SF group showed stronger FC expression in the brainstem (pons; Figure 4).

Overall, SF group demonstrated more localized FC patterns, primarily in ipsilateral mesial temporal and frontal regions, whereas NSF group showed more diffuse and contralateral FC, involving temporal, frontal, insular, subcortical and cerebellar regions (Table 2).

## Discussion

To the best of our knowledge, this is the first study to investigate pre-operative resting-state brain ICA-derived FC patterns from rs-fMRI and its association with LITT seizure outcomes. Significantly contributing spatial patterns, derived from ICA, were identified by applying a GLM to ICs within the cohort, adjusted for clinical covariates. The novelty of our study lies in its use of ICA-derived spatial patterns, which extend beyond traditional surgical targets to provide a whole-brain perspective on surgical outcomes. The reported significant results represent regions where the strength of ICA-derived functional connectivity (quantified by voxelwise *z*-scores within each IC) is systematically associated with surgical outcome across subjects. In other words, these maps identify brain regions where greater preoperative connectivity strength within meaningful ICs corresponds to a higher likelihood of seizure freedom. Thus, the “ICA-derived FC patterns” reflect spatially localized preoperative network features linked to surgical success, rather than averaged or aggregated IC activity.

Our findings revealed that mTLE patients with pre-operative brain ICA-derived FC patterns that was more locally distributed within the limbic network and brainstem were more likely to achieve seizure freedom. In contrast, patients exhibiting more diffusely distributed pattern, including connectivity involving the insula and basal ganglia, had decreased chances of SF outcomes. These results are noteworthy because they demonstrate that even without restricting analysis to predefined limbic networks, our data-driven approach still identified the importance of key limbic structures in predicting surgical outcomes. More broadly, this approach allowed us to detect cohort-specific network features within relevant brain regions that may not be fully captured by traditional region-of-interest strategies.

### Clinical and surgical factors

In this study, we included four clinical covariates. While meta-analyses of LITT for various types of TLE have reported higher rates of seizure freedom in patients with MTS (2, 24), a multicenter study focusing specifically on mTLE found no significant association between MTS and surgical outcomes beyond 12 months of follow-up (3). This finding is consistent with results from two other studies (25, 26). However, the presence of tonic-clonic seizures was negatively associated with seizure outcomes (3). We also included age at epilepsy onset as a covariate, as shorter epilepsy duration has been shown to be a positive prognostic factor (27, 28).

Our results also emphasize the advantage of focusing on global spatial patterns rather than ablation volume. Similar findings have been reported in prior functional connectivity (FC) studies of ATL for TLE, where the volume of surgical resection showed no difference between the SF and NSF groups (29). Instead, increased degree of local connectivity at the ipsilateral temporal pole and mesial temporal cortex was associated with post-surgical SF (30). Furthermore, previous studies have shown that preoperative rs-fMRI can reliably identify the epileptogenic zone, matching results from intracranial EEG in pediatric epilepsy patients (21). This study confirms the utility of pre-operative rs-fMRI as a valuable tool for predicting outcomes, and even planning ablation, without being influenced by intraoperative factors.

Ablation size and volume are often cited as important predictors of surgical success. However, these metrics can introduce bias, as surgical decisions may be subjective and physician-dependent. Our study deliberately focused on pre-operative spatial patterns to avoid potential bias and ensure that predictions were not influenced by intraoperative factors. This approach reflects a realistic clinical scenario in which pre-operative rs-fMRI data would be used to guide patient selection and predict outcomes without knowledge of the ablation size or location.

### Pre-operative resting state fMRI

Successful epilepsy surgery depends on the accurate localization and sufficient resection of EZ (14, 17). Previous LITT studies reported that ablation of mesial structures including the amygdala, hippocampus, parahippocampal gyrus, piriform cortex, and rhinal cortices, rather than ablation volume, was associated with higher rates of seizure freedom (3, 12, 31). Our findings align with these observations, suggesting that pre-operative resting-state brain FC patterns involving the anterior (vs. posterior) mesial temporal lobe (PHG and hippocampus) is more likely to achieve seizure freedom. Subjects with regions of FC patterns close to the ablation and within known limbic structures were more likely to have SF outcomes. Specifically, the bilateral posterior OFC, bilateral inferior frontal gyri, bilateral anterior superior temporal gyri, and ipsilateral middle temporal gyrus were areas in which SF subjects demonstrated spontaneous FC pattern.

Neural dysfunction in focal epilepsy has been increasingly viewed as a network disorder that extends beyond the EZ, supported by diminished global FC, as well as reduced FC within resting-state networks (e.g., default mode network), in contrast to healthy controls (32–35). Even when different types of surgery for TLE are considered, differences in preoperative global FC have been associated with seizure outcomes (17, 36, 37). Prior work from our group used atlas-based resting-state FC and found that the SF group was associated with increased coupling strength between the contralateral amygdala and contralateral precuneus, but decreased FC between brainstem and the ipsilateral thalamus (38). In the present study, we focused on the spatial pattern of spontaneous brain ICA-derived FC pattern (resting state BOLD) rather than the functional connectivity between predefined ROIs.

From a whole-brain perspective, our results showed that the distributions of brain FC pattern in the SF group were constrained relatively local near the mesial temporal cortex; but widespread to the temporooccipital and frontal lobes in NSF, namely the ipsilateral precuneus, lingual, and bilateral fusiform. Additionally, the NSF group had higher FC pattern in the bilateral insula cortices and the contralateral thalamus, indicating a diffusely distributed network. These spatial patterns suggest that a localized neural network, rather than diffuse brain FC pattern, may be more conducive to achieving seizure freedom.

Previous studies of TLE after temporal lobectomy (ATL or SAH) have commonly used graph theory analyses on preoperative rs-fMRI. Relative to the default mode network, the SF group showed increased centrality in the right anterior superior temporal gyrus, while the NSF group had higher centrality in the bilateral PCC/precuneus and inferior parietal lobe (39). In the analyses of the global network, a centralized integration in the contralateral temporo-insular region has been associated with SF (36), while increased centrality in the bilateral thalami associated with NSF (37). Studies have also suggested that the laterality of FC may serve as a predictor for seizure freedom. For FC seeded to the resected region, increased laterality is a predictor for better surgical outcomes (16); while for FC related to thalamus, increased laterality is a predictor for NSF (34, 40). Dynamic FC analysis has also been performed, in which NSF has been associated with more time spent in a low strength state and with a reduced number of transitions between states (33).

The more diffusely distributed ICA components in non–seizure-free (NSF) patients, encompassing thalamic and insular regions, may reflect a broader epileptogenic network beyond the temporal lobe. This interpretation is consistent with prior FDG-PET and EEG literature: patients with extratemporal hypometabolism on PET or bilateral/diffuse interictal discharges on EEG are less likely to achieve seizure freedom after temporal lobectomy (41–46). In contrast, focal ipsilateral abnormalities on both modalities predict more favorable outcomes. Taken together, these multimodal findings support the plausibility that diffuse ICA patterns in our cohort similarly mark an expanded epileptogenic network and lower surgical success.

We also observed that NSF patients exhibited higher contralateral activation patterns compared to the SF group. This likely reflects a more diffuse and less localized epileptogenic network that may not respond as effectively to focal interventions such as LITT. While some degree of contralateral involvement may represent adaptive or compensatory reorganization, as seen in the SF group, more extensive contralateral and extratemporal engagement, as shown in Table 2, suggests broader network dysfunction. Prior studies have similarly reported that widespread bilateral or extratemporal abnormalities, as well as contralateral network integration on presurgical rs-fMRI, PET, or EEG, are associated with poorer seizure outcomes after temporal surgery (33, 36, 42, 47, 48). Thus, contralateral alterations in SF patients may indicate functional compensation that does not preclude seizure freedom, whereas the more extensive contralateral activity observed in NSF patients likely reflects a less localizable and more distributed epileptogenic network that is less likely to respond well to LITT (33, 36, 42). These findings underscore the potential importance of the spatial distribution and laterality of functional network expression as predictors of surgical outcome, warranting validation in larger cohorts.

### Cerebellum and brainstem

Few studies have investigated the role of cerebellum and brainstem in TLE and surgical outcomes. TLE has been reported with diminished connectivity in the brainstem ascending reticular activating system (ARAS) (49) and atrophy in the posterior cerebellum (50) in contrast to healthy controls. Crossed cerebellar diaschisis in TLE, mainly identified through positron emission tomography (PET) and single photon emission tomography (SPECT) scans, has shown significant hypometabolism in the contralateral cerebellum associated with ipsilateral frontal lobe hypometabolism (51, 52). Regarding surgical outcomes, a reduction of total cerebellar gray matter volume was observed in the NSF group compared to the SF group (53). In the present study, brain ICA-derived FC pattern in the brainstem, specifically the pons, was significantly associated with SF. In cerebellum, multiple clusters in the bilateral posterior cerebellum were seen in both cohorts; but the contralateral anterior cerebellum (cerebellum 3, 4, and 5) was only seen in the NSF cohort. Our findings further highlight the underappreciated roles of brainstem and cerebellum in TLE and their potential in predicting surgical outcomes.

Overall, this study sheds new light on previously investigated FC patterns by demonstrating the importance of data-driven network extraction. Several AAL-defined ROIs contained separate clusters that distinctly contributed to the SF or NSF outcomes. Namely, the contralateral middle frontal gyrus and rectus (as part of the OFC) involves a posterior cluster that contributes to SF and an anterior cluster that contributes to NSF; the bilateral parahippocampal gyrus and the contralateral temporal superior gyrus both include an anterior cluster that associates with SF and a posterior cluster that associates with NSF; the contralateral post-central region involves an inferior cluster associating with SF and a superior cluster associating with NSF; and the contralateral cerebellum involves a medial cluster contributes to SF and a lateral cluster contributing to NSF. Our results highlight the utility of a data-driven approach for identifying clinically relevant spatial patterns, which may complement findings from traditional atlas-based analyses. These findings emphasize the potential of pre-operative rs-fMRI as a non-invasive tool for guiding patient selection and predicting outcomes in LITT for mTLE, offering valuable insights for clinical decision-making.

## Limitations

This study's retrospective design limited the sample size, as only a subset of patients met the inclusion criteria. However, all included data were complete, and we focused exclusively on mTLE to maintain consistency. We note that hemisphere-flipping may mask potential differences between left and right TLE. However, given the limited number of cases in each lateralized subgroup in the present cohort, formal left-only vs. right-only comparisons were underpowered. To minimize dataset exclusion and maximize sensitivity, we generated a homogeneous TLE group by flipping brains of right TLE patients (inverting x-axis coordinates). Thus, our rsfMRI results are referenced to the epileptogenic focus (ipsilateral/contralateral) rather than to fixed hemispheric location (left/right). This precludes the assessment of lateralization-specific FC patterns, which may influence surgical outcomes. This approach has been widely used in both structural and functional epilepsy (29, 54–56), with the recognized trade-off that potential lateralization effects may be reduced. A recent study found overall similar rs-fMRI patterns between left and right TLE compared to healthy controls, with no differences in voxel-mirrored homotopic connectivity (VMHC) (56). The study also identified increased patterns in bilateral TLE network regions (medial temporal lobe, thalamus, pons, etc.) and decreased patterns in the ipsilateral lateral temporal lobe. However, differences were also noted between left and right TLE, such as increased cerebellar patterns (in fractional ALFF and ReHo) and reduced ReHo in the right hippocampus. Lateralization-specific analyses in larger future cohorts will be needed to further confirm these findings.

Manual ICA component selection was used in this study to enable detailed evaluation of spatial and temporal features, which can be difficult for automated classifiers to capture in clinical populations. We acknowledge, however, that this approach introduces a degree of subjectivity. Future studies with larger datasets may benefit from automated or hybrid IC classification methods to enhance reproducibility. Another important methodological consideration is that the number and structure of ICs generated by ICA can vary depending on model order, pre-processing parameters (e.g., spatial smoothing), and scan length. In this study, we applied consistent pre-processing and automatic dimensionality estimation across all participants, and most retained ICs corresponded to canonical RSNs reproducibly identified across subjects. Nonetheless, the results may still be influenced by these analytic choices. Sensitivity analyses across different ICA dimensionalities, or the use of complementary approaches such as dual regression following group ICA or automated noise classifiers, would further strengthen reproducibility. Future studies with larger cohorts will be important to systematically compare these methods and to evaluate the robustness of patient-specific vs. group-level ICA frameworks, particularly in the context of predicting clinical outcomes such as post-surgical seizure control.

Another methodological limitation is the use of a linear regression model (i.e., an identity link) rather than a logistic or probit model for the binary outcome. This approach was chosen for its simplicity and subject-level interpretability within the voxelwise GLM framework and is consistent with prior applications in task-based and resting-state fMRI (57, 58) and lesion–symptom mapping (59–62). While this framework is established in neuroimaging, future studies may benefit from evaluating logistic or mixed-effects formulations to confirm the robustness of the observed associations. Lastly, this study compared the SF and NSF groups without including a healthy control group for reference. To clarify, the clusters of brain ICA-derived FC pattern identified in our study were in relation to each other group. When applying our results to clinical settings, it is essential to have some form of database or reference for comparison.

## Conclusion

This study utilized data-driven ICA and GLM to assess pre-operative resting-state brain ICA-derived FC pattern in mTLE and identify significant spatial patterns associated with seizure outcomes after LITT. We found that higher brain FC pattern that is locally distributed closer to the ipsilateral mesial temporal lobe, specifically the posterior OFC and anterior PHG, is associated with greater chance of seizure freedom. Conversely, more diffusely distributed brain FC pattern, particularly covering the insula and thalami, is more likely to have seizure recurrence. Our findings emphasize the importance of evaluating whole-brain networks, rather than focusing solely on EZ localization, in predicting outcomes. As the first study to explore data-driven spatial patterns contributing to LITT outcomes, we demonstrate the potential of pre-operative rs-fMRI as a non-invasive tool for patient selection. Future studies with larger datasets are warranted to validate our findings and assess their accuracy to enhance clinical implementation.

## Data Availability

The original contributions presented in the study are included in the article/Supplementary material, further inquiries can be directed to the corresponding author.
