# Supplementary material for: Independent component analysis of resting-state fMRI identifies regions associated with seizure freedom after laser interstitial thermal therapy for temporal lobe epilepsy
Source: Front Neurol. 2025 Nov 25;16:1675066. doi: 10.3389/fneur.2025.1675066 (PMC12685642; doi:10.3389/fneur.2025.1675066)
Supplement: Supplementary file 1 [file Table_1.docx]

Supplementary Table 1. Significant clusters associated with seizure free (SF) and non-seizure free (NSF).

|  | **Seizure Free (SF)**  24 significant clusters | | |  | **Not Seizure Free (NSF)**  34 significant clusters | | |
| --- | --- | --- | --- | --- | --- | --- | --- |
| **Area** | **Voxels (n)** | **Max (x, y, z)*** | **AAL brain regions** |  | **Voxels (n)** | **Max (x, y, z)*** | **AAL brain regions** |
| Frontal | 216 | (55, 89, 55) | Frontal_Sup_L, Frontal_Sup_Medial_L, Frontal_Sup_Medial_R |  | 101 | (50, 86, 26) | Frontal_Sup_Orb_L, Rectus_BL |
|  | 154 | (34, 77, 26) | Frontal_Sup_Orb_R, Frontal_Mid_Orb_R, Frontal_Inf_Orb_R, Olfactory_R, Rectus_BL |  | 49 | (39, 55, 75) | Precentral_R, Frontal_Sup_R, Supp_Motor_Area_R, Paracentral_Lobule_R |
|  | 78 | (51, 71, 65) | Frontal_Sup_L, Supp_Motor_Area_L, Frontal_Sup_Medial_L |  | 25 | (32, 80, 53) | Frontal_Mid_R |
|  | 68 | (56, 72, 26) | Frontal_Sup_Orb_L, Frontal_Inf_Orb_L |  | 24 | (61, 78, 53) | Frontal_Mid_L |
|  | 42 | (31, 73, 59) | Frontal_Sup_R, Frontal_Mid_R |  | 23 | (61, 76, 64) | Frontal_Mid_L |
|  | 38 | (73, 64, 56) | Precentral_L, Postcentral_L |  |  |  |  |
|  | 25 | (73, 72, 48) | Frontal_Inf_Oper_L, Frontal_Inf_Tri_L |  |  |  |  |
|  | 23 | (61, 56, 66) | Precentral_L, Frontal_Sup_L |  |  |  |  |
|  | 22 | (27, 74, 48) | Frontal_Mid_R, Frontal_Inf_Tri_R |  |  |  |  |
| Temporal | 65 | (75, 43, 34) | Temporal_Mid_L |  | 85 | (78, 52, 42) | Rolandic_Oper_L, Heschl_L, Temporal_Sup_L |
|  | 57 | (31, 59, 24) | Hippocampus_R, ParaHippocampal_R |  | 63 | (56, 49, 29) | ParaHippocampal_L, Lingual_L, Fusiform_L, Cerebelum_4_5_L |
|  | 43 | (72, 57, 29) | Temporal_Mid_L |  | 39 | (35, 45, 36) | Hippocampus_R, ParaHippocampal_R, Lingual_R |
|  | 38 | (71, 73, 27) | Temporal_Pole_Sup_L, Temporal_Pole_Mid_L |  | 32 | (60, 34, 31) | Lingual_L, Fusiform_L |
|  | 38 | (17, 61, 39) | Heschl_R, Temporal_Sup_R |  | 25 | (21, 44, 46) | Temporal_Sup_R |
|  | 30 | (59, 60, 24) | Hippocampus_L, ParaHippocampal_L |  | 22 | (32, 43, 31) | ParaHippocampal_R, Lingual_R, Fusiform_R |
|  | 24 | (50, 44, 40) | Cingulum_Post_L, Hippocampus_L, Precuneus_L |  | 20 | (61, 41, 33) | ParaHippocampal_L, Lingual_L, Fusiform_L |
|  |  |  |  |  | 20 | (77, 46, 47) | Temporal_Sup_L |
| Parietal | 36 | (19, 51, 63) | Postcentral_R, Parietal_Inf_R, SupraMarginal_R |  | 33 | (78, 46, 53) | SupraMarginal_L |
|  |  |  |  |  | 27 | (75, 41, 53) | SupraMarginal_L |
|  |  |  |  |  | 26 | (36, 49, 76) | Precentral_R, Postcentral_R |
|  |  |  |  |  | 21 | (76, 51, 57) | Parietal_Inf_L, SupraMarginal_L |
|  |  |  |  |  | 20 | (49, 27, 66) | Precuneus_L |
| Occipital | 26 | (33, 25, 60) | Cuneus_R, Occipital_Sup_R, Parietal_Sup_R |  | 50 | (59, 23, 51) | Occipital_Sup_L, Occipital_Mid_L |
|  |  |  |  |  | 23 | (58, 16, 29) | Lingual_L |
| Brainstem | 170 | (50, 55, 25) | Pons |  | - | - | - |
| Insula and | 45 | (47, 69, 34) | Olfactory_L, Olfactory_R, Caudate_BL |  | 21 | (26, 57, 36) | Insula_R, Temporal_Sup_R |
| Basal ganglia |  |  |  |  | 79 | (58, 63, 41) | Insula_L, Putamen_L, Pallidum_L |
|  |  |  |  |  | 69 | (32, 65, 39) | Putamen_R, Pallidum_R |
|  |  |  |  |  | 25 | (42, 56, 43) | Thalamus_R |
|  |  |  |  |  | 24 | (37, 56, 48) | Caudate_R |
|  |  |  |  |  | 22 | (38, 75, 36) | Caudate_R, Putamen_R |
| Cerebellum | 127 | (51, 29, 25) | Cerebelum_Crus1_L, Cerebelum_6_L, Vermis_6, Vermis_7 |  | 154 | (69, 33, 15) | Cerebelum_Crus1_L, Cerebelum_Crus2_L, Cerebelum_7b_L, Cerebelum_8_L |
|  | 53 | (26, 30, 24) | Cerebelum_Crus1_R, Cerebelum_6_R |  | 54 | (30, 34, 30) | Fusiform_R, Cerebelum_4_5_R, Cerebelum_6_R |
|  | 23 | (48, 30, 20) | Cerebelum_Crus1_L, Cerebelum_Crus2_L, Cerebelum_8_L, Vermis_8 |  | 50 | (57, 35, 10) | Cerebelum_8_L, Cerebelum_9_L |
|  | 21 | (48, 39, 12) | Cerebelum_9_L |  | 37 | (41, 42, 26) | Cerebelum_3_R, Cerebelum_4_5_R, Vermis_1_2, Vermis_3 |
|  |  |  |  |  | 33 | (47, 40, 36) | Lingual_L, Cerebelum_4_5_L, Vermis_4_5 |
|  |  |  |  |  | 23 | (42, 42, 12) | Cerebelum_9_R |
|  |  |  |  |  | 22 | (30, 41, 24) | Cerebelum_4_5_R, Cerebelum_6_R |
|  |  |  |  |  | 21 | (20, 37, 20) | Cerebelum_Crus1_R |
| *Coordinates of the maximum t-score are index (x, y, z) with origin at the right, posterior, inferior corner of image.  Abbreviations: L = left/ipsilateral, R = right/contralateral, BL = bilateral | | | | | | | |
